# Spontaneous Mandibular Dentoalveolar Changes after Rapid Maxillary Expansion (RME), Slow Maxillary Expansion (SME), and Leaf Expander—A Systematic Review

**DOI:** 10.3390/children11040501

**Published:** 2024-04-22

**Authors:** Alessandro Ugolini, Andrea Abate, Margherita Donelli, Francesca Gaffuri, Alessandro Bruni, Cinzia Maspero, Valentina Lanteri

**Affiliations:** 1 Department of Sciences Integrated Surgical and Diagnostic, University of Genova, 16145 Genova, Italy; alessandro.ugolini@unige.it; 2 Department of Biomedical Surgical and Dental Sciences, University of Milan, 20129 Milan, Italy; margherita.donelli@unimi.it (M.D.); francesca.gaffuri@unimi.it (F.G.); cinzia.maspero@unimi.it (C.M.); 3 Fondazione IRCCS Cà Granda, Ospedale Maggiore Policlinico, 20142 Milan, Italy; 4 Surgical, Medical and Dental Department, University of Modena and Reggio Emilia, 41125 Modena, Italy; alebruni@unimore.it (A.B.); valentina.lanteri@unimore.it (V.L.)

**Keywords:** malocclusions, maxillary hypoplasia, maxillary expansion, spontaneous mandibular changes

## Abstract

Background: This systematic review aims to analyze the spontaneous dentoalveolar changes in the mandibular arch after maxillary expansion in growing patients obtained with different expansion protocols: Rapid Maxillary Expansion (RME), Slow Maxillary Expansion (SME), and Leaf Expander. Methods: The study adhered to the Preferred Reporting Items for Systematic Review and Meta-Analysis (PRISMA) guidelines. Eligibility criteria were established in the PICO format, involving patients who underwent slow, rapid, or leaf maxillary expansion during the mixed or early permanent dentitions. A comprehensive search of electronic databases and manual searches was conducted up to December 2023. The outcome measures included inter-mandibular first permanent molar width, inter-deciduous molar and canine width, arch perimeter, and arch length; both short- and long-term results were considered. The articles that met the inclusion criteria were included in this systematic review and were qualitatively evaluated using a methodological quality scoring system with a 13-point scale. To assess the inter-examiner agreement concerning the article selection and the qualitative assessment of the included studies, Kappa statistics were computed. Results: A total of 1184 articles were identified through electronic and manual searches. After the removal of duplicates and the initial examination of the titles and abstracts, 57 articles were considered for the full text analysis, and according to the eligibility and exclusion criteria, 22 studies were finally selected, composed of 8 randomized controlled trials (RCTs) and 14 retrospective/case-control studies. The qualitative assessment of the included studies showed the following scores: 6 papers have high research quality, 5 have moderate quality, and 11 have low quality. SME demonstrated negligible mandibular changes, with less than 1 mm variation on average (range 0.46–2.00 mm) in the selected parameters and relapses observed in the long term. RME induced more significant increases, particularly in intermolar width greater than 1 mm, which ranged between 0.93 and 3.3 mm, and good stability over the long term. Leaf Expander exhibited promising short-term lower intermolar width increases greater than 1 mm and ranged from 0.5 to 1.69 mm, but long-term stability was not thoroughly evaluated. Conclusions: SME results in negligible short- and long-term effects, while RME, especially with Haas-type appliances, exhibits significant intermolar width increases that remain stable over the years. Leaf Expander shows short-term lower intermolar width increases, requiring further investigation into long-term stability.

## 1. Introduction

For over a century, the combination of orthopedic and orthodontic tooth movements has been used to correct palatal transverse deficiency. It is commonly associated with crowding in the upper arch, nasal airway obstruction, and unilateral or bilateral posterior crossbite during the mixed or early permanent dentitions [1,2,3].

If left untreated, posterior crossbite occasionally produces mandibular shift or postural alterations and asymmetrical growth of the mandible, or dysfunction of the skeletal and muscle structures [4,5].

Rapid maxillary expansion (RME) is the most commonly used orthopedic therapy for the correction of this condition in growing patients. It increases the palatal transverse dimension, creating additional space for the dental arch to correct upper crowding through the separation of the mid-palatal suture [6].

The significance of interceptive treatment during the early mixed dentition phase lies in its ability to create sufficient space, facilitating proper tooth eruption. Rapid maxillary expansion can be obtained with different devices, such as tooth–tissue-borne appliances (Haas expander) and tooth-borne appliances (Hyrax expander, also known as Biederman expander) [7]. Tooth-borne maxillary expanders rely solely on the teeth for anchorage, often utilizing bands or attachments bonded directly to the teeth; in contrast, tooth–tissue-borne maxillary expanders employ both the teeth and surrounding oral tissues for anchorage, distributing expansion forces more evenly.

Expansion of the maxilla can be achieved through the use of either slow or rapid expansion appliances, provided that the median palatine suture has not yet completed its skeletal maturation. However, once sutural fusion occurs, surgical intervention becomes necessary for expansion [8]. Surgically-assisted rapid palatal expansion (SARPE) is the preferred approach for orthopedic expansion in non-growing adolescents and adults, as it effectively reduces resistance in the sutures. SARPE reliably widens the maxilla and serves as a highly efficient method for skeletal expansion, particularly in patients with fully fused midpalatal sutures [8,9,10,11,12].

**Figure 1:**
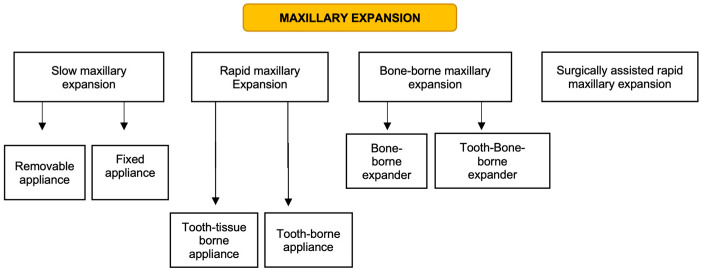
Maxillary expansion protocols.

Recent advancements in orthodontic treatment have introduced the use of skeletal anchorage appliances such as miniscrews and miniplates, revolutionizing the design of maxillary expansion devices [13]. These innovative appliances aim to achieve skeletal expansion while minimizing adverse effects on dental structures. Studies have documented successful maxillary expansion with miniscrew-supported appliances, underscoring their ability to widen the maxillary arch without significant repercussions on teeth and periodontal health [9,14]. Currently, bone-borne expansion appliances stand out as the preferred option for reducing side effects in non-growing adolescents and adults. Figure 1 summarizes the different types of maxillary expansion.

Considering that maxillary constriction can eventually result in mandibular dental arch constriction, maxillary expansion may potentially trigger a spontaneous increase in mandibular dental arch width, either in the short or long term. This phenomenon occurs due to the modification of force equilibrium between the tongue and cheek on the mandibular teeth following maxillary dental arch expansion [15]. In 1961, Haas [16,17,18,19] noticed that, when a maxillary expansion of 12–14 mm was performed, an evident spontaneous dentoalveolar expansion would occur in the lower arch.

An altered balance between the tongue and buccinator muscles, with an increased predominance of tongue forces exerted on the mandibular teeth, could potentially lead to an augmentation in mandibular dental arch width [20,21].

Spontaneous dentoalveolar changes occurring in the mandibular dental arch concurrently with slow maxillary expansion (SME) or rapid maxillary expansion (RME) may carry clinical implications. Recent studies utilizing 3D non-invasive analysis have provided evidence supporting the indirect effects of RME on the mandibular arch [22,23,24]. The authors reported significant changes at the level of the mandibular dental arch, confirming that this modification is a significant effect to be considered during the treatment. Behnamour et al. [25] reported in a recent study that the increase in the arch perimeter of the lower arch after maxillary expansion was considered negligible. The authors also stated that a greater mandibular intermolar width occurred in patients treated with Leaf Expander, as previously reported by other researchers [22].

Although maxillary expansion treatment has been widely described in the literature, mandibular arch spontaneous decompensation after RME is not completely certain with state-of-the-art evidence.

Mandibular dental changes following tooth-borne maxillary expansion obtained with slow or rapid maxillary expansion have been compared in different studies with contradictory results.

Until now, only one systematic review has been published in the literature with the aim of analyzing this parameter [26].

Furthermore, despite the recent introduction of a new palatal expander featuring Ni–Ti leaf springs (Leaf Expander, Leone, Italy) designed to produce lower, constant, and calibrated forces for expansion, no systematic review has been proposed to compare the effects of the Leaf Expander with other expansion protocols concerning the mandibular dental changes. The literature highlighted similar results comparing Leaf Expander protocols and the RME expanders [6,24,27,28,29,30,31,32,33] and a lower level of pain [34] during the initial days following the application of the Leaf Expander. Thus, the purpose of this systematic review is to perform a close investigation to evaluate the spontaneous dentoalveolar changes after different maxillary expansion protocols in growing subjects during mixed or early permanent dentitions.

## 2. Materials and Methods

### 2.1. Protocol and Registration

This systematic review protocol was pre-registered with the National Institute of Health Research Database (http://www.crd.york.ac.uk/prospero, Protocol I.D. CRD 42021283294, 4 November 2021).

The review was conducted based on the Preferred Reporting Items for Systematic Review and Meta-Analysis (PRISMA 2020 checklist) statement [35] (See Supplementary Materials).

### 2.2. Eligibility Criteria

The inclusion criteria included patients with mixed or early permanent dentition, healthy children, or young adults requiring maxillary expansion. Additionally, the criteria encompassed patients treated with both slow and rapid maxillary expansion techniques and studies that provided clear descriptions of the types of appliances utilized. The exclusion criteria encompassed studies involving clefts and/or palate or other craniofacial anomalies; interventions in the mandibular dental arch during the follow-up period; any non-tooth-borne protocols of maxillary expansion; as well as studies lacking a control group. The criteria to deem the study eligible to be included in the current systematic review were defined in the PICO format and are listed in Table 1.

### 2.3. Information Source

A thorough electronic search of data was systematically conducted up to December 2023 without imposing any restriction on the year of publication. Five databases were searched, including Medline (via PubMed), Medline (via Ovid), Embase (via Ovid), Web of Science, Cochrane Library, and ClinicalTrials.gov. Manual research was also performed, screening the reference list of the eligible studies and review articles to find additional proper articles. No place or publication date restrictions were utilized, but only English papers were included in this review.

Moreover, unpublished studies were retrieved by searching in trial registries (ClinicalTrials.gov) and evaluating the databases of grey literature (Open Grey).

### 2.4. Search Strategy

A search strategy using database-specific controlled text (MeSH terms) and predefined fields was adopted to find relevant articles. A query string was generated for the PubMed (MEDLINE) database research and then modified according to the PICOS format for the other databases.

After recovering all the results from different databases, they were imported and merged into a specific screening and data extraction tool, the Rayyan web application (https://www.rayyan.ai). This software was used to automatically remove the duplicates, and after that, a hand screening was carried out to make sure that no duplicate references remained.

### 2.5. Selection Progress

After removing duplicate references, the references of the ultimately included articles were reviewed for pertinent content. Additionally, the reference lists of eligible articles were manually scrutinized to identify other potentially relevant studies. Two independent investigators performed the search (F.G. and A.A.). Initially, the examiners independently screened the titles and abstracts of the studies retrieved from each database, following the Participants, Interventions, Comparisons, Outcomes, and Study (PICOS) design scheme. The evaluation of titles and abstracts was conducted utilizing the Rayyan web application (https://www.rayyan.ai), which facilitated the search process [36]. In instances of uncertainty regarding eligibility, the full texts of those articles that met the eligibility criteria and suggested that they could be related to the purpose of this systematic review were retrieved and read independently in duplicate by the same reviewers. Any disagreements on eligibility were resolved through discussion with a third author (M.D.).

### 2.6. Data Collection Process and Data Items

The data extraction process from the articles evaluated for eligibility was conducted independently by the same two reviewer authors (A.A. and F.G.) in duplicate, and disagreements were handled by a discussion with a third reviewer (M.D.).

The information collected for the included studies encompassed the following details: authors and year of publication, study design, number of participants, mean age of the patients, sample size calculation, intervention, reported outcomes of interest, and treatment period. Numerical data were extracted and rounded to two decimals wherever feasible; in cases where this was not possible, data were recorded exactly as reported by the included papers.

Concerning the outcomes of interest, at least the following variables by means of digital dental models were examined: inter-deciduous canine width (C-C), inter-deciduous molar width (E-E), inter-first permanent molar width (6-6), arch perimeter, and arch length [37].

When the intermolar distances were measured both at the lingual and buccal cusp tips of the teeth, the lingual values were chosen to analyze the mandibular transversal width changes. It was found that the methods used to measure the bucco–lingual inclination of the canines and the mandibular molars were too heterogeneous, so this parameter was not included in the results.

### 2.7. Study Risk of Bias Assessment

The articles meeting the inclusion criteria were incorporated into this systematic review and subjected to qualitative evaluation following the Cochrane Collaboration recommendations, adopting the modification developed by Vilani et al. [38] related to the methodological quality score scale in order to evaluate both randomized and non-randomized trials (Table 2). The qualitative assessment was performed independently by two investigators (F.G. and M.D.), and disagreements were resolved after discussion with a third author (A.A.).

### 2.8. Dealing with Missing Data

Missing data were obtained by contacting the corresponding authors of the included studies. Otherwise, the Cochrane Handbook recommendations were followed.

### 2.9. Summary of Measures and Data Synthesis

Data were summarized, and a comprehensive qualitative synthesis of the results was performed, including the predetermined outcomes of this review. Inter-author reliability concerning the article selection and the qualitative assessment was assessed as a percentage of agreement using Cohen’s Kappa statistics. Moreover, a meta-analysis was not undertaken because of the heterogeneity of the included studies. The assessment of clinical heterogeneity involved examining the appliances utilized, the timing of outcome measurements, and the participants’ age and malocclusion characteristics.

### 2.10. Certainty Assessment

The quality of the evidence was assessed using the Grading of Recommendations, Assessment, Development, and Evaluations (GRADE) approach.

## 3. Results

A total of 1184 articles were discovered through both electronic and manual searches (see Appendix A). Detailed electronic search strategies utilized for each database are provided in the same appendix. After eliminating duplicate entries and conducting an initial assessment of titles and abstracts, 57 articles were shortlisted.

**Figure 2:**
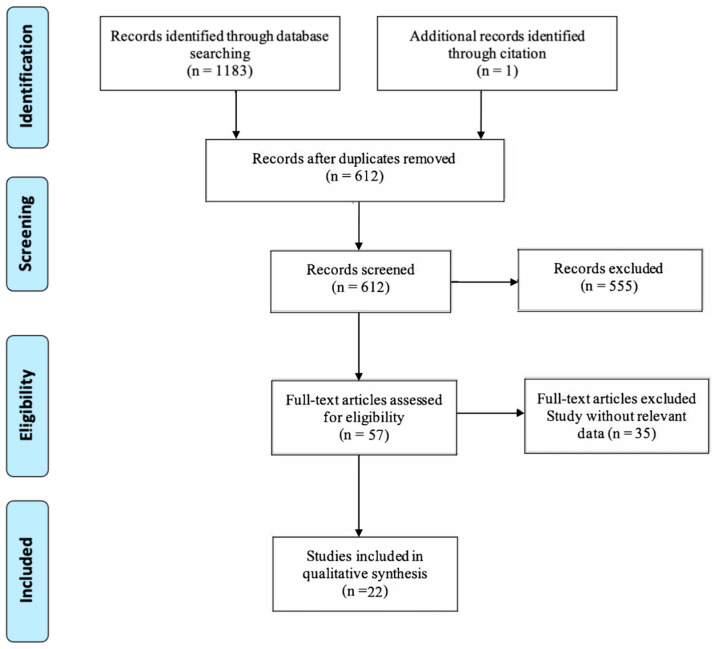
Flow diagram adapted from PRISMA 2020 displaying the quantity of records identified and excluded at each phase of the review.

The full text of these articles was thoroughly reviewed against the eligibility and exclusion criteria, resulting in the inclusion of 22 studies for the final analysis (refer to Appendix B). A flowchart illustrating the process of article selection is presented in Figure 2.

The Kappa statistic was performed after article selection in order to evaluate the agreement between the investigators and showed excellent inter-examiner agreement (K = 0.94). A summary of the methodological investigation about all the results of the mandibular changes found in the selected articles involving study design, number of treated patients, mean ages, expansion procedure, type of appliances, amount of expansion, follow-up period, and lower arch measurements is shown in Table 2 and Table 3.

The Kappa statistic was also calculated after the studies’ quality assessment, indicating a good inter-examiner agreement (K = 0.85).

Eight studies were RCT; the others had a retrospective or a case-control clinical design.

Table 4 reports the methodological quality score scale applied to the eight articles included in order to evaluate both randomized and non-randomized trials.

More than half of the authors studied the dental effects in the lower arch using RME.

The spontaneous changes were assessed in both the short term (3 to approximately 12 months post-expansion) and long term (more than 12 months post-expansion). All of the included papers evaluated the changes in the mandibular dental arch following either slow or rapid maxillary expansion or the use of the Leaf Expander. Measurements were performed using digital sliding calipers on dental models. The principal variables examined across these investigations included: inter-deciduous canine width (C-C), inter-deciduous molar width (E-E), inter-first permanent molar width (6-6), arch perimeter, and arch length.

Table 4 summarizes all the variables analyzed in each study along with the relative amount of maxillary expansion. 

### 3.1. SME and Spontaneous Mandibular Changes

Three authors [28,44,49] investigated short-term mandibular changes (3–12 months post-expansion) after different devices with a SME protocol. Petrén et al. [49] enrolled 35 subjects treated with the SME protocol and observed a slight increase in transversal measures for both the quad helix (QH) (C-C = +0.2 mm, 6-6 = −0.4 mm) and the removable-palate (RP) expanders (C-C = +0.6 mm, 6-6 = +0.4 mm).

Otherwise, two articles highlighted significant increases in intercanine and intermolar width (>1 mm) after QH treatment [44] and the ELA SME appliance [28].

Four articles [45,46,47,52] investigated both short- and long-term (more than 12 months post-expansion) lower arch spontaneous changes after SME with RP or QH. The results showed little or no changes in the lower arch after 1 year of treatment. Moreover, after 8 months from the end of the expansion, all authors showed similar arch relapses or no significant changes in the lower intercanine and intermolar width.

In particular, Godoy et al. [45] found that the QH group had greater intermolar expansion (+0.46 mm) than the RP group (−0.12 mm) after crossbite resolution, a stable result (+0.46 mm) for the QH group after 16 months, and a further decrease in intermolar width for the RP group (−0.36 mm). A similarly designed study [52] observed no changes in lower intermolar and intercanine width after 7 months of treatment, and a mild decrease in lower intercanine and an unchanged intermolar width were observed about 6 years after the end of treatment, both using RP and QH as the SME activation protocols.

### 3.2. RME and Spontaneous Mandibular Changes

Eleven articles [22,23,24,25,28,29,32,39,41,44,48] investigated short-term mandibular spontaneous effects after RME protocols. The long-term mandibular spontaneous changes evaluation [20,40,42,50,51] after RME was made by five authors, two of whom, Cozzani et al. [50] and O’Grady et al. [51], also analyzed short-term results. In a randomized controlled trial (RCT) [23] on 48 patients, both the expander with differential opening (EDO) and the fan-type expander (FE) induced mild spontaneous changes in the mandibular arch after 6 months of RME. The EDO showed a slightly greater increase in intermolar distance (0.93 ± 0.91 mm) compared to the FE (0.12 ± 0.89 mm). Both expanders caused very small widening of the mandibular dental arch without a perimeter arch increase, resulting in an equal decrease in mandibular arch length.

Another RCT [39] on 16 patients treated with RME (Hyrax expander) found no differences for mandibular interdental width after approximately 2 weeks of activation (0.18 ± 0.46 mm for C-C and 0.37 ± 0.42 mm for 6-6) but a significant increase (+0.71 ± 0.56 mm) in the intermolar distance after 6 months of retention. In subjects treated with a Haas expander, after 11 months of RME treatment, the mandibular intermolar width significantly increased by 2.02 mm, the primary intermolar width increased by 1.75 mm, the intercanine width increased by 0.9 mm, and the total arch length increased by 0.7 mm [22]. Another study investigated the same appliance [50], with an approximately similar amount of maxillary expansion, and determined superimposable spontaneous mandibular changes. After 1 year, there was an increase in mandibular intercanine and inter-deciduous molar distances of 0.9 mm and 0.7 mm, respectively; the values for canines remained stable after the 13 months of follow-up. However, this paper showed no statistically significant treatment-induced or longitudinal changes in the intermolar width after RME. Likewise, Ugolini et al. [40], who observed subjects treated with a Haas expander and did an analogous follow-up period (15 months), found a similar result both for intercanine width (+1 mm) and mandibular intermolar width (+0.7 mm). One author [41] compared patients treated with RME (Haas expander) anchored to the first permanent molars (Gr6) with those treated with RME anchored to the first deciduous molars (GrE). They found a general tendency toward expansion of the lower arch (+1.8 mm for C-C and +1.4 mm for 6-6) in Gr6 after 1 year of treatment, but always a stable situation (C-C and 6-6 = +0.5 mm expansion) after 1 year in GrE. Another author [43] obtained results for RME (Hyrax-type) both 6 months and 1 year after treatment and found a bigger expansion for C-C and 6-6 (1.14 mm and 2.12 mm, respectively) in the shorter-term follow-up than after 1 year (0.81 mm for C-C and 1.65 mm for 6-6). A case-control study [42] assessing the mandibular changes after two different treatment protocols of rapid maxillary expansion (RME and MME) found at T1 (1.3 ± 0.2 years) an increase in mandibular intermolar and intercanine distance (*p* < 0.05). This increase is higher in the MME group, both for intercanine (RME: +0.85; MME: +1.13) and intermolar (RME: +1.5; MME: +2.09) width. Lima et al. [20] analyzed 30 subjects treated with RME and, after 12 years of follow-up, observed a significant (*p* = 0.001) decrease in arch length (−4.30 mm) and arch perimeter (−4.85 mm). Additionally, there was a significant increase in mandibular intermolar width of 0.93 mm from pre-expansion to long-term follow-up (*p* < 0.05). The intercanine width decreased by −0.99 mm (*p* = 0.001). Similarly, after approximately 1 year from the start of the treatment with an acrylic-bonded maxillary expander [51], an increase of 2.1 mm for the intermolar width that remained +1.6 mm after 3 years with a significant decrease of 3.6 mm in arch perimeter was observed.

### 3.3. Leaf Expander and Spontaneous Mandibular Changes

Five authors [24,25,28,29,32] analyzed the spontaneous mandibular changes following maxillary expansion using the Leaf Expander protocol by comparing it with the results obtained from RME and SME protocols.

In further detail, Paoloni et al. [24] evaluated the spontaneous mandibular changes after 1 year for both RME and the Leaf Expander, with similar results of approximately 0.5 mm of changes obtained with both methods. Benhamour et al. [25] examined the effects of different maxillary expansion techniques on the lower arch. It was observed that RME and the Leaf Expander had similar increases in arch perimeter on the order of 0.3 mm, which were not statistically significant. The Leaf Expander, however, produced a significant increase in the intermolar width, 1.54 ± 0.82 mm, and a strong correlation between mandibular tipping and Leaf Expander expansion was observed. Cossellu et al. [32] reported a statistically significant difference in all the variables analyzed (*p* < 0.05). In particular, the mandibular interdental width increased significantly by 1.24 mm (SD 1.09 mm).

Abate et al. [29] studied the Leaf Expander and RME effects of the lower arch, observing a statistically significant increase in lower intermolar width in both groups with no significant differences in other mandibular dentoalveolar parameters.

Lanteri et al. [28] investigated the changes after RME, SME, and Leaf Expander, finding significant improvements in maxillary and mandibular dimensions. The average mandibular width increases varied across the techniques: 3.3 ± 4.4 mm with the RME, 2.0 ± 1.7 mm with the SME, and 1.4 ± 1.6 mm with the Leaf Expander. Table 5 summarizes the findings of the review.

## 4. Discussion

Maxillary dental arch constriction is a common occurrence, often seen alongside unilateral or bilateral posterior crossbites, particularly during the mixed dentition phase or early permanent dentition stage [53,54,55,56,57,58,59].

The presence of mandibular changes after palatal expansion has been reported since 1970 by clinical studies [60,61,62,63,64,65].

The dentoskeletal effects, both short-term and long-term, of slow and rapid palatal expansion have already been investigated and documented in systematic reviews and meta-analyses, but none of these evaluated the effects on the lower dental arch [5,66,67,68,69,70].

Mandibular dentoalveolar effects with slow and rapid maxillary expansion have already been analyzed by means of radiographs and three-dimensional methods by several authors [45,46,49,50,51,52]. However, only one previous systematic review [71] evaluated the mandibular effects obtained after RME or SME, which included six articles.

Our review, which is more recent, assessed the short-term and long-term spontaneous mandibular response after maxillary expansion, comparing RME, SME, and Leaf Expander protocols and analyzing 22 articles with a more thorough look at the results.

The selected studies showed negligible mandibular changes of less than 1 mm for patients treated with SME.

Patients treated with QH or RP had similar results after the crossbite correction, with small differences.

Petren et al. [46] found that 35 subjects treated with QH obtained smaller increases in intercanine and intermolar width than those treated with RP. The same authors [49], 3 years earlier, found slightly different results with a 12-month follow-up period. Godoy et al. [45] found that the QH group had greater intermolar expansion (0.46 mm) than the RP group (−0.36 mm).

Bjerklin et al. [52], a decade earlier, recorded similar non-significant results for mandibular changes in both the short and long term using QH and RP. The primary findings by these authors indicated that, compared to QH, treatment duration with RP was nearly twice as long, potentially explaining why transversal dimensions were smaller in the QH group compared to the RP group in the long term.

Conversely, Shundo et al. [44] showed that the QH treatment increased the intermolar (+1.44 mm) and inter-deciduous molar (+1 mm) width. The long-term outcomes observed in their study contradicted those reported in the aforementioned study, which could potentially be attributed to changes in occlusal forces following maxillary expansion.

Additionally, no significant changes were found by Wong et al. [47] for all mandibular measurements obtained after the initial treatment effects of three types of expanders activated with SME, although 4 mm of total buccal tipping occurred between the molars.

The clinical significance of these findings suggests that SME may not lead to clinically significant spontaneous dentoalveolar changes. Instead, the papers that investigated the short- and long-term effects of RME found more interesting outcomes.

Ugolini et al. (2015) [41], in contrast to previous studies [20,72], did not identify a statistically significant increase in lower intermolar width. However, they did observe a notable increase in molar angulation within the rapid maxillary expansion (RME) group, where anchorage was provided at the first permanent molars, after one year. This alteration may have been influenced by occlusal changes resulting from the molar angulation increase. As a result, it is probable that the resultant force vector exerted on the mandibular teeth was oriented more towards the vestibular aspect. This observation aligns with the theory proposed by Haas, wherein the occlusal aspect of the lingual cusp of the upper first molars comes into contact with the occlusal aspect of the facial cusp of the lower first molars [73].

Grassia et al. [43] found that the mandibular intermolar arch width increased 1.65 mm per year and the mandibular intercanine width increased 0.81 mm per year (*p* < 0.001), more than the results reported of about 1 mm by other authors [23,24,39].

The same authors [42] published research one year later that found similar results with statistically significant increases (*p* < 0.05) in mandibular intermolar width of +1.5 mm and increases in intercanine arch width of +0.85. In addition, they found slightly greater increases in the same values after treatment with MME (mixed maxillary expansion): +1.13 mm for intercanine distances and +2.09 mm for intermolar distances.

Lima et al., in a long-term retrospective study, investigated patients treated with RME and found increases in mandibular intermolar width (0.97 mm) and in intercanine width (0.26 mm) in the 1 year follow-up, with no statistically significant changes for arch length and arch perimeter.

The augmentation in intermolar width indicates a subtle uprighting effect. To gauge the extent of alterations potentially linked to natural growth, the occlusal width measurements of each individual were juxtaposed with Moorees’ average width adjustments for each corresponding side, factoring in the child’s age and gender [74]. Consequently, it was determined that 0.08 mm of the mandibular intermolar width increase could be attributed to changes through normal growth, while 1.39 mm (out of 1.47 mm) was attributed to rapid maxillary expansion (RME).

Scant data are available regarding the impacts of the Leaf Expander on the mandibular arch. Nevertheless, initial results indicate a rise of 0.49 mm in the lower molars, signifying an innate adjustment of the lower dentition [32]. Additionally, the downward displacement of the tongue resulting from the presence of the expander (the Leaf Expander shares a similar structure with the RME of the control group) influences and facilitates the expansion in the alignment of the lower teeth [32].

A marginal disparity, devoid of statistical significance, was noted in the lower arch perimeter (LAP), which expanded by less than 1 mm in both the Leaf Expander and RME cohorts. These results are consistent with those documented in earlier scientific literature [22,75].

Concerning the reason behind the effect of maxillary expansion on the lower arch, as reported by Abate et al. [29], the augmentation in intermolar width could be ascribed to increased tongue pressure prompted by the presence of the appliance, resulting in a downward displacement of the tongue, reduced lip and cheek pressures, and the formation of new occlusal contacts. Importantly, occlusal interactions between the palatal cusp of the upper first molars and the buccal cusp of the lower first permanent molars may contribute to these changes.

Another biomechanical explanation of the slightly spontaneous mandibular changes after maxillary expansion is the “lip bumper effect”, previously described by Haas [76]: As upper transversal widths progressively expanded, especially during the long-term follow-up phase characterized by the shift from mixed to permanent dentition, it was noted that the cheeks moved further away from the buccal surfaces of the mandibular teeth. Consequently, the lower teeth experienced uprighting. This uprighting phenomenon was more pronounced in the molar region compared to the canine area, attributed to greater posterior upper arch changes.

Thus, one possibility is that the lateral movement of the maxillary arch enlarged the area of attachment of the buccal muscles, resulting in an expansion of the lower arch too.

The minimal widening of the mandibular dental arch observed in several studies, without significant changes in arch perimeter or mandibular arch length [20,23,47], is likely attributed to dental development during the mixed dentition phase, which results in the loss of leeway space [77,78].

### Limitations

The limitations of this systematic review stem from the limited inclusion of randomized controlled trials (RCTs), which may compromise the depth and robustness of the evidence synthesized, highlighting the need for additional high-quality RCTs to strengthen future analyses in this domain.

Further prospective investigations utilizing CBCT, incorporating appropriate sample sizes and extensive long-term monitoring, are essential to quantitatively evaluating skeletal and dentoalveolar changes post-treatment with the Leaf Expander and RME. Nevertheless, the unwarranted application of CBCT, owing to the risks associated with ionizing radiation, is strongly discouraged, particularly in pediatric patients, as indicated by the DIMITRA guidelines [79] and recommendations from the British Orthodontic Society and the American Association of Orthodontists [80,81,82,83,84,85]. The ongoing evolution of 3D radiation-free imaging modalities, such as magnetic resonance imaging (MRI), shows promise in addressing this concern in forthcoming studies [86,87,88,89,90,91].

Improvements in the methodological rigor of studies and the uniformity of RCTs hold promise for yielding further insights in future meta-analyses.

This has the potential to produce elevated levels of scientific evidence, consequently reinforcing orthodontic clinical practice. In summary, additional RCTs are imperative to comprehensively evaluate the impacts of maxillary expansion on the lower dental arch. By addressing existing literature gaps, these studies hold promise for delivering more resilient evidence for informed clinical decision-making in orthodontics.

## 5. Conclusions

Based on the results of this systematic review, it can be concluded that the three maxillary expansion protocols analyzed—RME, SME, and Leaf Expander—can induce changes in the mandibular arch with heterogeneous entities.

Negligible short- and long-term spontaneous dentoalveolar changes were observed in the parameters of the mandibular dental arch following slow maxillary expansion (SME). However, a relapse of the lower arch length and perimeter was noted in the long term, with relatively high confidence in the evidence. Conversely, more significant changes were observed after rapid maxillary expansion (RME) in the mixed and early permanent dentition stages, with a significant increase in mandibular intermolar width post-RME that remained stable over the year. It is important to note that these findings are based on low confidence in the evidence.

Also, for Leaf Expander, a significant spontaneous increase in lower intermolar width was seen in the short-term follow-up; on the other hand, no Leaf Expander study has evaluated the long-term results’ stability.

In summary, the effects may vary based on the type of expander used and the follow-up duration. To better understand the behavior of the mandibular arch after maxillary expansion, more RCTs with a long-term assessment are needed, particularly for RME and Leaf Expander protocols.

## Figures and Tables

**Table 1:** The inclusion and exclusion criteria adopted for the study selection.

Category	Inclusion Criteria	Exclusion Criteria
Participants	- Studies on growing patients with mixed or early permanent dentition - Studies with homogeneous groups of patients with maxillary transverse deficiency (as defined by the authors of the individual primary studies)	- In vitro studies - Studies on animals - Studies on adults - Studies concerning clefts and/or palate or other craniofacial anomalies - Studies including patients with systemic disease or craniofacial syndrome - Intervention in the mandibular dental arch during the follow-up period
Intervention	- Participants receiving a slow or rapid maxillary expansion with a Hyrax expander (also known as a Biederman appliance), quad helix, Removable-Plate, or Haas expander - Participants receiving a slow maxillary expansion with the Leaf Expander	- Orthodontic treatment in the lower arch - Any other maxillary expander
Comparison	- Participants treated with different types of appliances - Different treatment phases - Untreated controls	- Studies without comparison
Outcomes	- Primary outcome: mandibular intermolar distance - Secondary outcome: mandibular intercanine distance	- Studies reporting different clinical outcomes
Types of included studies	- Randomized controlled clinical trials (RCTs) and non-randomized studies (NRS) - Prospective and retrospective NRS	- Other reviews - Studies without a comparative group - Letter to the editor or commentaries - Non-English articles - Case reports/case series

**Table 2:** Characteristics of the included studies.

Author	Year	Study Design	Sample	Mean Age (y)	Expansion Procedure	Appliance	Amount of exp. (mm)	Follow-up Period	Measurements
Paoloni et al. [24]	2022	RCT	56	SME: 8 ± 1.3 RME: 8.4 ± 1.0	SME RME	Leaf Expander Hyrax expander	4.5	1 year	C-C, 6-6
Massaro et al. [23]	2021	RCT	48	7.62	RME	EDO FE	8	6 months	C-C, E-E, 6-6, Arch length and perimeter
Di Ventura et al. [22]	2019	CC study	21	7.4 ± 1.2	RME	Haas expander	6.59 ± 1.28	11 months	C-C, E-E, 6-6
Canan et al. [39]	2017	RCT	16	12.63 ± 1.36	RME	Hyrax expander	7.07 ± 1.25	T1: 13.3 ± 2.78 d T2: 6.76 ± 0.53 m	3-3, 6-6
Ugolini et al. [40]	2016	CC study	33	8.8 ± 1.1	RME	Haas expander	Not mentioned	15 months	C-C, 6-6
Ugolini et al. [41]	2015	RCT	70	8.4 ± 1.1	RME	GrE Gr6	GrE: 9 ± 1.76 Gr6: 7.7 ± 1.32	T1 ~6 months T2 11/12 months	C-C, 6-6
Grassia et al. [42]	2015	CC study	42	RME: 8.8 ± 1.37 MME: 8.9 ± 2.34	RME MME	Hyrax expander	Not mentioned	RME: 1.2 ± 0.3 years MME: 1.3 ± 0.2 years	C-C, 6-6
Grassia et al. [43]	2014	CC study	24	8.6 ± 2.0	RME	Hyrax expander	2 mm overcorrection	T1: 6 ± 2 m T2: 1 years	C-C, 6-6
Shundo et al. [44]	2012	CC study	50	9.5 ± 1.7	SME	Quad Helix	Not mentioned	1 year ± 8 months	E-E, 6-6
Godoy et al. [45]	2011	RCT	66	QH: 8 ± 0.79 RP: 7.82 ± 0.85	SME	Quad Helix Removable-Plate	Not mentioned	T1: until crossbite correction (4.5 m) T2: 6 months after retention	C-C, 6-6
Petrén et al. [46]	2011	RCT	35	QH: 9.0 ± 1.19 RP: 8.5 ± 1.02	SME	Quad Helix Removable-Plate	Not mentioned	T1: 6 months T2: 3 years	C-C, 6-6
Wong et al. [47]	2011	RCT	110	7 ± 7	SME	• Haas-type acrylic appliance • Hyrax expander • Quad Helix	2 mm overcorrection	T1: 1 ± 1 month T2: 4 years	C-C, 6-6, Arch length
Santos et al. [48]	2010	Retrosp. study	21	7.6–16.5 years	RME	Hyrax expander	6.27 mm	After removing the Expander	6-6
Petrén et al. [49]	2008	RCT	30	QH: 9.1 ± 1.03 RP: 8.7 ± 0.82	SME	• Quad Helix • Removable-Plate	Not mentioned	1 year	C-C, 6-6
Cozzani et al. [50]	2007	Retrosp. study	31	7.3 years ± 12 months	RME	Haas expander	6.8 mm	T1: 1.1 years ± 4 m T2: 2.4 years ± 1.7 m	C-C, E-E, 6-6
O’Grady et al. [51]	2006	Retrosp. study	27	8.5 ± 1.3	RME	Acrylic-bonded maxillary expander	7–8 mm	T1: 9–14 months T2: 3.2 years	C-C, 6-6, Arch length and perimeter
Lima et al. [20]	2004	Retrosp. study	30	8.2	RME	Palatal expander	8–11 mm	T1: 1.2 years T2: 5 years T3: 12.5 years	C-C, 6-6, Arch length and perimeter
Bjerklin et al. [52]	2000	Longitud. study	38	QH: 9.3 ± 1.39 RP: 9.2 ± 1.52	SME	• Quad Helix • Removable-Plate	Not mentioned	T1: QH 12.5 m, RP 7.7 m T2: QH 82 m, RP 76 m	C-C, 6-6
Benhamour et al. [25]	2022	Retrosp. Study	54	HEX:10.5 ± 1.7 LEX:9.8 ± 1.5	RME LEAF	• Hyrax expander • Leaf Expander (900 gr)	HEX: 5.53 ± 1.19 mm LEX: 5.18 ± 0.81 mm	T1-T0: 4 months	C-C, 6-6, E-E, arch perimeter
Cossellu et al. [32]	2020	Retrosp. study	90	7.5 y ± 1.5	RME LEAF	• Haas expander • Leaf Expander (6 mm–450 gr)	6 mm	9–11 months	C-C, 6-6, E-E
Abate et al. [29]	2023	Retrosp. study	47	RME: 8.2 ± 0.8 LE: 7.9 ± 0.7	RME LEAF	• Hyrax expander • Leaf Expander (6/9 mm–450 gr)	RME: 10 mm LE: 6–9 mm	RME: 8.6 months LE: 9.4 months	C-C, 6-6, Arch perimeter
Lanteri et al. [28]	2018	Retrosp. study	30	RME: 8.9 y ± 1.6 SME: 12.2 y ± 2.4 LE: 7.11 y ± 1.3	RME SME LE	• Haas expander • ELA • Leaf Expander	RME: not ment SME: not ment LE: 6 mm	RME: 7 months SME: 10 months LE: 11 months	6-6

**Table 3:** Summarized data from the included studies.

Author	Exp	Amount of Expansion	FU	C-C	6-6	E-E	Arch Length	Arch Perimeter
Paoloni et al. [24]	RME + Leaf	4.5 mm	1 year	Leaf: 0.7 ± 1.0 RME: 0.3 ± 1.0	Leaf: 0.5 ± 1.2 RME: 0.5 ± 1.1	Leaf: 0.5 ± 1.2 RME: 0.5 ± 1.1	/	/
Massaro et al. [23]	RME	8 mm	6 months	EDO: −0.35 ± 1.1 FE: −0.05 ± 0.67	EDO: 0.93 ± 0.91 FE: 0.12 ± 0.89	EDO: 0.59 ± 0.66 FE: 0.31 ± 0.79	EDO: −0.55 ± 0.6 FE: −0.52 ± 0.6	EDO: −0.64 ± 0.9 FE: −0.66 ± 1.3
Di Ventura et al. [22]	RME	6.59 ± 1.28	11 months	+0.95 ± 1.1	+2.02 ± 1.43	+1.75 ± 1.38	/	+0.72 ± 1.2
Canan et al. [39]	RME	7.07 ± 1.25	T1: 13.3 ± 2.78 d T2: 6.76 ± 0.53 m	T1: +0.18 ± 0.46 T2: +0.32 ± 0.51	T1: +0.37 ± 0.42 T2: +0.71 ± 0.56	/	/	/
Ugolini et al. [40]	RME	Not mentioned	15 months	+1.0	+0.7	/	/	/
Ugolini et al. [41]	RME	GrE: 9 ± 1.76 Gr6: 7.7 ± 1.32	T1: 6 months T2: 11/12 months	GrE: 0.4, Gr6: 1.6 GrE: 0.5, Gr6: 1.8	GrE: 0.5, Gr6: 0.6 GrE: 0.5, Gr6: 1.4	/	/	/
Grassia et al. [42]	RME MME	Not mentioned	RME: 1.2 ± 0.3 years MME: 1.3 ± 0.2 years	RME: 0.85 MME: 1.13	RME: 1.5 MME: 2.09	/	/	/
Grassia et al. [43]	RME	2 mm overcorrection	T1: 6 ± 2 m T2: 1 year	+1.14 +0.81	+2.12 +1.65	/	/	/
Shundo et al. [44]	SME	Not mentioned	1 year ± 8 months	/	1.44 ± 1.32	1 ± 2.25	/	/
Godoy et al. [45]	SME	Not mentioned	T1: until crossbite correction (4.5 m) T2: 6 months after retention	QH: 0.05 ± 1.66 RP: 0.39 ± 1.56 QH: −0.21 ± 0.92 RP: 0.28 ± 1.51	QH: 0.46 ± 1.23 RP: −0.36 ± 1.71 QH: 0.46 ± 1.2 RP: −0.12 ± 1.36	/	/	/
Petrén et al. [46]	SME	Not mentioned	T1: 6 months T2: 3 years	QH: 0.2 ± 1.05 RP: 0.6 ± 1.63 QH: −1 ± 1.1 RP: −1.8 ± 1.4	QH: −0.4 ± 0.82 RP: 0.4 ± 0.67 QH: −0.2 ± 0.92 RP: −1 ± 1.15	/	/	/
Wong et al. [47]	SME	2 mm overcorrection	T1: 1 y ± 1 month T2: 4 years	−0.19 ± 0.26 −0.35 ± 0.25	0.27 ± 0.56 0.66 ± 0.56	/	−0.65 ± 0.30 −2.26 ± 0.30	/
Santos et al. [48]	RME	6.27 mm	Unspecified	/	+0.34	/	/	/
Petrén et al. [49]	SME	Not mentioned	1 year	QH: 0.1 ± 0.26 RP: 0.2 ± 0.28	QH: −0.1 ± 0.62 RP: 0.5 ± 0.67	/	/	/
Cozzani et al. [50]	RME	6.8 mm	T1: 1.1 years ± 4 months T2: 2.4 years ± 1.7 months	0.9 ± 2.1 0.9 ± 2.3	0.2 ± 2.4 0.1 ± 2.4	0.7 ± 2.6 0.1 ± 2.6	/	/
O’Grady et al. [51]	RME	7–8 mm	T1: 9–14 months T2: 3.2 years	0.1 ± 1.8	2.1 ± 1.8 1.6 ± 1.7	/	/	−1.2 ± 2.2 −3.6 ± 3.2
Lima et al. [20]	RME	8–11 mm	T1: 1.2 years T2: 5 years T3: 12.5 years	0.39 ± 0.81 −0.68 ± 1.14 −0.99 ± 1.22	0.97 ± 0.88 1.05 ± 1.41 0.93 ± 1.77	/	/ −2.85 ± 1.88 −4.3 ± 1.71	/ −3.33 ± 2.66 −4.85 ± 1.83
Bjerklin et al. [52]	SME	Not mentioned	T1: QH 12.5, RP 7.7 months T2: QH 81.9, RP 76.1 months	T1: QH: 0.1 ± 0.23 RP: −0.1 ± 0.91 T2: QH: −0.9 ± 1.15 RP: −0.4 ± 0.99	T1: QH: 0.0 ± 0.21 RP: 0.0 ± 0.57 T2: QH: 0.5 ± 0.88 RP: −0.1 ± 0.64	/	/	/
Benhamour et al. [25]	RME LEAF	HEX: 5.53 ± 1.19 mm LEX: 5.18 ± 0.81 mm	T1-T0: 4 months	HEX: 0.36 ± 0.74 LEX: 0.53 ± 0.84	HEX: 0.49 ± 0.77 LEX: 1.54 ± 0.82	HEX: 0.42 ± 0.77 LEX: 1.30 ± 0.95	/	HEX: 0.37 ± 1.08 LEX: 0.32 ± 0.85
Cossellu et al. [32]	RME LEAF	6 mm	9–11 months	RME: 0.95 ± 1.1 LE: 1.03 ± 1.25	RME: 2.02 ± 1.33 LE: 1.24 ± 1.9	RME: 1.75 ± 1.38 LE: 1.63 ± 1.57	/	/
Abate et al. [29]	RME LEAF	RME: 10 mm LE: 6–9 mm	RME: 8.6 months LE: 9.4 months	RME: 0.25 ± 0.97 LE: 0.16 ± 0.72	RME: 2.14 ± 0.87 LE: 1.69 ± 1.07	/	/	RME: 0.65 ± 2.20 LE: 0.34 ± 2.64
Lanteri et al. [28]	RME SME LEAF	RME: not ment. SME: not ment. LE: 6 mm	RME: 7 months SME: 10 months LE: 11 months	/	RME: 3.3 ± 4.4 SME: 2.0 ± 1.7 LE: 1.4 ± 1.6	/	/	/

**Table 4:** Quality assessment of the selected studies.

	Selection Bias		Performance Bias	Detection Bias	Attrition Bias	Reporting Bias	Eligible Criteria for Participants	Presence of Control Group	Other Kinds of Bias			Total Points	Research Quality
Article	Randomization	Allocation Concealment	Blinding of Participants	Blinding Assessment	Incomplete Outcome	Selective Reporting			Statistical Treatment	Reliability of Measures	Potential Bias and Trial Limitations		
Paoloni et al. [24]	0.5	0.5	1	1	1	0.5	1	1	1	1	1	9.5	High
Massaro et al. [23]	0.5	0.5	0.5	1	1	0.5	1	1	1	1	1	9	High
Di Ventura et al. [22]	0.5	0	0	0	1	0.5	1	1	1	0	1	6	Moderate
Canan et al. [39]	0.5	0	0	0	0	0.5	1	0	1	0	0	3	Low
Ugolini et al. [40]	0	0	0	0	0	0.5	1	1	1	0	0	3.5	Low
Ugolini et al. [41]	1	1	0.5	1	0	0.5	1	0	1	1	0	7	Moderate
Grassia et al. [42]	0	0	0	0	1	0.5	1	0	1	0	0.5	4	Low
Grassia et al. [43]	0	0	0.5	0	0.5	0.5	1	0	1	0	0	3.5	Low
Shundo et al. [44]	1	0	1	1	1	1	1	1	1	1	0	9	High
Godoy et al. [45]	1	1	0	1	0.5	1	1	1	1	1	1	9.5	High
Petrén et al. [46]	0.5	0.5	0	1	1	0.5	1	1	1	1	1	8.5	High
Wong et al. [47]	0	1	0.5	0.5	1	0.5	1	0.5	1	0.5	0.5	7	Moderate
Santos et al. [48]	0	0.5	0	0	0.5	0.5	1	0	1	0	0.5	4	Low
Petrén et al. [49]	1	1	0	1	1	1	1	1	1	1	1	10	High
Cozzani et al. [50]	0	0.5	0	0	0.5	0.5	0.5	1	0.5	1	0	4.5	Low
O’Grady et al. [51]	0	0.5	0	0	0.5	0.5	1	1	0.5	1	0	5	Low
Lima et al. [20]	0	0	0	0	1	0.5	1	0	1	0	0	3.5	Low
Bjerklin et al. [52]	0	0.5	0	0	0.5	0.5	1	1	0.5	1	0	5	Moderate
Benhamour et al. [25]	0	0	0	0	0.5	0.5	1	0	1	0	0	3	Low
Cossellu et al. [32]	0	0	0	0	0.5	0.5	1	0	1	0	0	3	Low
Abate et al. [29]	0	0.5	0	0	0.5	0.5	1	0.	0.5	1	0	5	Moderate
Lanteri et al. [28]	0	0	0	0	1	0.5	1	0	1	1	0	4.5	Low

The quality of research or methodological robustness: high, >8 points; moderate, 5 to 8 points; low, <5 points.

**Table 5:** Summary of the review findings.

Maxillary Expansion Method	Mean Values			Studies Contributing to the Review Finding	Qualitative Assessement	Explanation of the Qualitative Assessment
SME: The selected studies showed negligible mandibular changes of less than 1 mm for patients treated with SME, both in the short- and long-term evaluations.	C-C	E-E	6-6	Reference	High confidence in the evidence	Four studies scored high [44,45,46,49], two moderate [47,52], and one low [28]
	/	/	2.0 ± 1.7	[28]		
	/	1 ± 2.25	1.44 ± 1.32	[44]		
	0.05 ± 1.66	/	0.46 ± 1.23	[45]		
	−1 ± 1.1	/	−0.2 ± 0.92	[46]		
	−0.35 ± 0.25	/	0.66 ± 0.56	[47]		
	0.1 ± 0.26	/	−0.1 ± 0.62	[49]		
	−0.9 ± 1.15	/	0.5 ± 0.88	[52]		
RME: Relevant changes occur after RME in the mixed and early permanent dentition. Mandibular intermolar width increased significantly after RME and remained stable for years (>1 mm).	C-C	E-E	6-6	[8,9,10,11,18,20,23,27,32,33,34]	Low confidence in the evidence	10 studies scored low [20,25,28,32,39,40,42,48,50,51], 3 moderate [22,29,41], and 3 high [23,24,44]
	−0.99 ± 1.22		0.93 ± 1.77	[20]		
	+0.95 ± 1.1	+1.75 ± 1.38	+2.02 ± 1.43	[22]		
	−0.35 ± 1.1	0.59 ± 0.66	0.93 ± 0.91	[23]		
	0.3 ± 1.0	0.5 ± 1.1	0.5 ± 1.1	[24]		
	0.36 ± 0.74	0.42 ± 0.77	0.49 ± 0.77	[25]		
	/	/	3.3 ± 4.4	[28]		
	0.25 ± 0.97	/	2.14 ± 0.87	[29]		
	0.95 ± 1.1	1.75 ± 1.38	2.02 ± 1.33	[32]		
	+0.32 ± 0.51	/	+0.71 ± 0.56	[39]		
	+1.0 ± 0.1	/	+0.7 ± 0.2	[40]		
	1.8 ± 0.4	/	1.4 ± 0.3	[41]		
	0.85		1.5	[42]		
	/	/	1.44 ± 1.32	[44]		
	/	/	+0.34	[48]		
	0.9 ± 2.3	/	0.1 ± 2.4	[50]		
	0.1 ± 1.5	/	1.6 ± 1.7	[51]		
Leaf Expander: There was a significant spontaneous increase in lower intermolar width in the short-term follow-up (>1 mm); no Leaf Expander study evaluated the long-term results’ stability.	C-C	E-E	6-6	[8,9,10,11,18,20,23,27,32,33,34]	Moderate confidence in the evidence	Three studies scored low [25,28,32], one moderate, [29], and one high [24]
	0.7 ± 1.0	0.5 ± 1.2	0.5 ± 1.2	[24]		
	0.53 ± 0.84	1.30 ± 0.95	1.54 ± 0.82	[25]		
	/	/	1.4 ± 1.6	[28]		
	0.16 ± 0.72	/	1.69 ± 1.07	[29]		
	1.03 ± 1.25	1.63 ± 1.57	1.24 ± 1.9	[32]		

## Data Availability

This systematic review protocol was registered in advance with the National Institute of Health Research Database (http://www.crd.york.ac.uk/prospero, Protocol I.D. CRD 42021283294).

## Supplementary Materials

The following supporting information can be downloaded at: https://www.mdpi.com/article/10.3390/children11040501/s1, PRISMA 2020 Checklist. Reference [92] is cited in the supplementary materials.

## Appendix A. Search String for Each Database

**Database**	**Search Strategy**	**Results**
PubMed/Medline	(“Palatal Expansion Technique”[Mesh] OR (Palatal expander*) OR (Hyrax expander) OR (Leaf expander) OR (Quad Helix) OR (“maxillary expander”) OR (removable expander) OR (Schwarz expander)) AND ((Mandibular response*) OR (mandibular decompensation) OR (mandibular change*) OR (mandibular effect*) OR (lower arch response*) OR (lower arch decompensation) OR (lower arch change*) OR (lower arch effect*) OR (dentoalveolar change*) OR (dentoalveolar effect*))	611
Cochrane Library	#1 MeSH descriptor: [Palatal Expansion Technique] explode all trees #2 (Palatal expander*) OR (Hyrax expander) OR (Leaf expander) OR (Quad Helix) OR (“maxillary expander”) OR (removable expander) OR (Schwarz expander) #3 #1 OR #2 #4 ((Mandibular response*) OR (mandibular decompensation) OR (mandibular change*) OR (mandibular effect*) OR (lower arch response*) OR (lower arch decompensation) OR (lower arch change*) OR (lower arch effect*) OR (dentoalveolar change*) OR (dentoalveolar effect*)) #5 #1 AND #4	61
Embase	#1 p‘alatal expansion’/exp OR p‘alatal expansion’ OR (palatal AND expander*) OR h‘yrax expander’ OR ((h‘yrax’/exp OR hyrax) AND expander) OR l‘eaf expander’ OR ((l‘eaf’/exp OR leaf) AND expander) OR q‘uad helix’/exp OR q‘uad helix’ OR ((q‘uad’/exp OR quad) AND (h‘elix’/exp OR helix)) OR m‘axillary expander’ OR r‘emovable expander’ OR (removable AND expander) OR s‘chwarz expander’ OR (schwarz AND expander) #2 mandibular AND response* OR (mandibular AND decompensation) OR (mandibular AND change*) OR (mandibular AND effect*) OR (lower AND arch AND response*) OR (lower AND arch AND decompensation) OR (lower AND arch AND change*) OR (lower AND arch AND effect*) OR (dentoalveolar AND change*) OR (dentoalveolar AND effect*) #1 AND #2	324
Web of Science	#1 ALL = ((Palatal expander*) OR (Hyrax expander) OR (Leaf expander) OR (Quad Helix) OR (“maxillary expander”) OR (removable expander) OR (Schwarz expander)) #2 ALL = (((Mandibular response*) OR (mandibular decompensation) OR (mandibular change*) OR (mandibular effect*) OR (lower arch response*) OR (lower arch decompensation) OR (lower arch change*) OR (lower arch effect*) OR (dentoalveolar change*) OR (dentoalveolar effect*))) #3 #1 AND #2	179
Other bibliographic databases	ClinicalTrials.gov Grey literature (opengrey)	8
A manual search	Reference lists of potentially included studies	1
Total		1184

## Appendix B. List of Full Text Exclusions with Reasons

**Study**	**Reasons for the Exclusion**
Koç O, Koç N, Jacob HB. Effect of different palatal expanders with miniscrews in surgically assisted rapid palatal expansion: A non-linear finite element analysis. Dental Press J Orthod. 2024 Mar 4;29(1):e2423195. doi: 10.1590/2177-6709.29.1.e2423195.oar. PMID: 38451569; PMCID: PMC10914319.	Outcomes of interest are not presented
Barone S, Bennardo F, Diodati F, Salviati M, Calabria E, Colangeli W, Antonelli A, Giudice C, Giudice A. Short- and Long-Term Effects of Maxillary Expander with Tongue Crib in Growing Open-Bite and Skeletal Class II Patients: A Retrospective Study. Dent J (Basel). 2024 Jan 24;12(2):22. doi: 10.3390/dj12020022. PMID: 38392226; PMCID: PMC10887548.	Outcomes of interest are not presented
Colino-Gallardo P, Del Fresno-Aguilar I, Castillo-Montaño L, Colino-Paniagua C, Baptista-Sánchez H, Criado-Pérez L, Alvarado-Lorenzo A. Skeletal and Dentoalveolar Changes in Growing Patients Treated with Rapid Maxillary Expansion Measured in 3D Cone-Beam Computed Tomography. Biomedicines. 2023 Dec 13;11(12):3305. doi: 10.3390/biomedicines11123305. PMID: 38137526; PMCID: PMC10740967.	Outcomes of interest are not presented
Wang C, Liu C, Mao Q, Zhou L, Xiang X. Skeletal and dentoalveolar modifications in adults with different sagittal facial patterns after personalized miniscrew-assisted rapid palatal expansion: A prospective cone-beam computed tomography study. Am J Orthod Dentofacial Orthop. 2023 Dec;164(6):843-854. doi: 10.1016/j.ajodo.2023.05.033. Epub 2023 Aug 26. PMID: 37632488.	Outcomes of interest are not presented
Takagi T, Tanaka E. An adult case of unilateral posterior crossbite caused by maxillary transverse deficiency treated with miniscrew-assisted rapid palatal expansion. J Stomatol Oral Maxillofac Surg. 2023 Dec;124(6):101443. doi: 10.1016/j.jormas.2023.101443. Epub 2023 Mar 16. PMID: 36933657.	Case report
Teixeira R, Massaro C, Garib D. Vertical and sagittal changes produced by an expander with differential opening and fan-type expander: A post-hoc analysis of a randomised controlled trial. J Orthod. 2023 Oct 31:14653125231208465. doi: 10.1177/14653125231208465. Epub ahead of print. PMID: 37905906.	Outcomes of interest are not presented
Zhang Y, Yang J, Li X. Assessment of early dental arch growth modification with removable maxillary expansion by cone-beam computed tomography and lateral cephalometric radiographs: a retrospective study. BMC Oral Health. 2023 Oct 7;23(1):727. doi: 10.1186/s12903-023-03433-w. PMID: 37805525; PMCID: PMC10559620	No outcomes available
Ning R, Chen J, Liu S, Lu Y. Treatment effects after maxillary expansion using tooth-borne vs tissue-borne miniscrew-assisted rapid palatal expansion appliance. Am J Orthod Dentofacial Orthop. 2023 Oct;164(4):545-553. doi: 10.1016/j.ajodo.2023.02.022. Epub 2023 May 13. PMID: 37178105.	Different appliances from those included in the review
Pasqua BPM, André CB, Paiva JB, Rino Neto J. Short-term assessment of pain and discomfort during rapid maxillary expansion with tooth-bone-borne and tooth-borne appliances: randomized clinical trial. Dental Press J Orthod. 2023 Sep 15;28(4):e2322220. doi: 10.1590/2177-6709.28.4.e2322220.oar. PMID: 37729286; PMCID: PMC10508049.	Outcomes of interest are not presented
Leonardi RM, Aboulazm K, Giudice AL, Ronsivalle V, D’Antò V, Lagravère M, Isola G. Evaluation of mandibular changes after rapid maxillary expansion: a CBCT study in youngsters with unilateral posterior crossbite using a surface-to-surface matching technique. Clin Oral Investig. 2021 Apr;25(4):1775-1785. doi: 10.1007/s00784-020-03480-5. Epub 2020 Aug 2. PMID: 32743674.	Outcomes of interest are not presented
Lione R, Fusaroli D, Mucedero M, Paoloni V, Pavoni C, Cozza P. Changes in mandibular shape after early treatment in subjects with open bite: a geometric morphometric analysis. Eur J Orthod. 2020 Dec 2;42(6):643-649. doi: 10.1093/ejo/cjz104. PMID: 31942983.	Outcomes of interest are not presented
Lo Giudice A, Ronsivalle V, Lagravere M, Leonardi R, Martina S, Isola G. Transverse dentoalveolar response of mandibular arch after rapid maxillary expansion (RME) with tooth-borne and bone-borne appliances. Angle Orthod. 2020 Sep 1;90(5):680-687. doi: 10.2319/042520-353.1. PMID: 33378488; PMCID: PMC8032272.	Different appliances from those included in the review
Evangelista K, Ferrari-Piloni C, Barros LAN, Avelino MAG, Helena Soares Cevidanes L, Ruellas ACO, Valladares-Neto J, Silva MAG. Three-dimensional assessment of craniofacial asymmetry in children with transverse maxillary deficiency after rapid maxillary expansion: A prospective study. Orthod Craniofac Res. 2020 Aug;23(3):300-312. doi: 10.1111/ocr.12370. Epub 2020 Feb 26. PMID: 32022986; PMCID: PMC7783112.	Outcomes of interest are not presented
Shen T, Zhao B, Wang C, Xiao Y, Han Y, Zhao G, Ke J. Efficacy of different designs of mandibular expanders: A 3-dimensional finite element study. Am J Orthod Dentofacial Orthop. 2020 May;157(5):641-650. doi: 10.1016/j.ajodo.2019.05.019. PMID: 32354437.	Different appliances from those included in the review
Lo Giudice A, Fastuca R, Portelli M, Militi A, Bellocchio M, Spinuzza P, Briguglio F, Caprioglio A, Nucera R. Effects of rapid vs slow maxillary expansion on nasal cavity dimensions in growing subjects: a methodological and reproducibility study. Eur J Paediatr Dent. 2017 Dec;18(4):299-304. doi: 10.23804/ejpd.2017.18.04.07. PMID: 2938061	Outcomes of interest are not presented
Lione R, Brunelli V, Franchi L, Pavoni C, Quiroga Souki B, Cozza P. Mandibular response after rapid maxillary expansion in class II growing patients: a pilot randomized controlled trial. Prog Orthod. 2017 Nov 6;18(1):36. doi: 10.1186/s40510-017-0189-6. Erratum in: Prog Orthod. 2018 Jul 12;19(1):26. PMID: 29105023; PMCID: PMC5673058.	Outcomes of interest are not presented
Cassi D, Di Blasio A, Gandolfinini M, Magnifico M, Pellegrino F, Piancino MG. Dentoalveolar Effects of Early Orthodontic Treatment in Patients With Cleft Lip and Palate. J Craniofac Surg. 2017 Nov;28(8):2021-2026. doi: 10.1097/SCS.0000000000003854. PMID: 28891894; PMCID: PMC5673300.	Different conditions from those included in the review
Alves ACM, Maranhão OBV, Janson G, Garib DG. Mandibular dental arch short and long-term spontaneous dentoalveolar changes after slow or rapid maxillary expansion: a systematic review. Dental Press J Orthod. 2017 May-Jun;22(3):55-63. doi: 10.1590/2177-6709.22.3.055-063.oar. PMID: 28746488; PMCID: PMC5525446.	Systematic review
Pereira JDS, Jacob HB, Locks A, Brunetto M, Ribeiro GLU. Evaluation of the rapid and slow maxillary expansion using cone-beam computed tomography: a randomized clinical trial. Dental Press J Orthod. 2017 Mar-Apr;22(2):61-68. doi: 10.1590/2177-6709.22.2.061-068.oar. PMID: 28658357; PMCID: PMC5484271.	Outcomes of interest are not presented
Manni A, Pasini M, Giuca MR, Morganti R, Cozzani M. A retrospective cephalometric study on pharyngeal airway space changes after rapid palatal expansion and Herbst appliance with or without skeletal anchorage. Prog Orthod. 2016 Dec;17(1):29. doi: 10.1186/s40510-016-0141-1. Epub 2016 Sep 26. PMID: 27641421; PMCID: PMC5035718	Outcomes of interest are not presented
Conroy-Piskai C, Galang-Boquiren MT, Obrez A, Viana MG, Oppermann N, Sanchez F, Edgren B, Kusnoto B. Assessment of vertical changes during maxillary expansion using quad helix or bonded rapid maxillary expander. Angle Orthod. 2016 Nov;86(6):925-933. doi: 10.2319/112315-799. Epub 2016 May 16. PMID: 27182780; PMCID:	Outcomes of interest are not presented
Raucci G, Pachêco-Pereira C, Elyasi M, d’Apuzzo F, Flores-Mir C, Perillo L. Short- and long-term evaluation of mandibular dental arch dimensional changes in patients treated with a lip bumper during mixed dentition followed by fixed appliances. Angle Orthod. 2016 Sep;86(5):753-60. doi: 10.2319/073015-519.1. Epub 2016 Jan 15. PMID: 26771718; PMCID: PMC8600827.	Different appliances from those included in the review
de Medeiros Alves AC, Garib DG, Janson G, de Almeida AM, Calil LR. Analysis of the dentoalveolar effects of slow and rapid maxillary expansion in complete bilateral cleft lip and palate patients: a randomized clinical trial. Clin Oral Investig. 2016 Sep;20(7):1837-47. doi: 10.1007/s00784-015-1675-1. Epub 2015 Dec 1. PMID: 2662	Different conditions from those included in the review
Halicioğlu K, Yavuz I. A comparison of the sagittal and vertical dentofacial effects of maxillary expansion produced by a memory screw and a hyrax screw. Aust Orthod J. 2016 May;32(1):31-40. PMID: 27468589.	Outcomes of interest are not presented
Taddei M, Alkhamis N, Tagariello T, D’Alessandro G, Mariucci EM, Piana G. Effects of rapid maxillary expansion and mandibular advancement on upper airways in Marfan’s syndrome children: a home sleep study and cephalometric evaluation. Sleep Breath. 2015 Dec;19(4):1213-20. doi: 10.1007/s11325-015-1141-y. Epub 2015 Feb 15. PMID: 25	Outcomes of interest are not presented
Gunyuz Toklu M, Germec-Cakan D, Tozlu M. Periodontal, dentoalveolar, and skeletal effects of tooth-borne and tooth-bone-borne expansion appliances. Am J Orthod Dentofacial Orthop. 2015 Jul;148(1):97-109. doi: 10.1016/j.ajodo.2015.02.022. PMID: 26124033.	Outcomes of interest are not presented
Venancio F, Alarcon JA, Lenguas L, Kassem M, Martin C. Mandibular kinematic changes after unilateral cross-bite with lateral shift correction. J Oral Rehabil. 2014 Oct;41(10):723-9. doi: 10.1111/joor.12199. Epub 2014 Jun 3. PMID: 24894509	Outcomes of interest are not presented
Perillo L, De Rosa A, Iaselli F, d’Apuzzo F, Grassia V, Cappabianca S. Comparison between rapid and mixed maxillary expansion through an assessment of dento-skeletal effects on posteroanterior cephalometry. Prog Orthod. 2014 Jul 18;15(1):46. doi: 10.1186/s40510-014-0046-9. PMID: 25139110; PMCID: PMC4138550.	Outcomes of interest are not presented
Lione R, Franchi L, Cozza P. Does rapid maxillary expansion induce adverse effects in growing subjects? Angle Orthod. 2013 Jan;83(1):172-82. doi: 10.2319/041012-300.1. Epub 2012 Jul 23. PMID: 22827478; PMCID: PMC8805530.	Systematic review
Gungor AY, Türkkahraman H, Baykul T, Alkis H. Comparison of the effects of rapid maxillary expansion and surgically assisted rapid maxillary expansion in the sagittal, vertical, and transverse planes. Med Oral Patol Oral Cir Bucal. 2012 Mar 1;17(2):e311-9. doi: 10.4317/medoral.17389. PMID: 22143686; PMCID: PMC3448316.	Different appliances from those included in the review
Domann CE, Kau CH, English JD, Xia JJ, Souccar NM, Lee RP. Cone beam computed tomography analysis of dentoalveolar changes immediately after maxillary expansion. Orthodontics (Chic.). 2011 Fall;12(3):202-9. PMID: 22022691; PMCID: PMC4638317.	Outcomes of interest are not presented
Wong CA, Sinclair PM, Keim RG, Kennedy DB. Arch dimension changes from successful slow maxillary expansion of unilateral posterior crossbite. Angle Orthod. 2011 Jul;81(4):616-23. doi: 10.2319/072210-429.1. Epub 2011 Feb 9. PMID: 21306221; PMCID: PMC8919736.	Outcomes of interest are not presented
Petrén S. Correction of unilateral posterior crossbite in the mixed dentition. Studies of treatment effects, stability and cost-effectiveness. Swed Dent J Suppl. 2011;(212):11-83. PMID: 21919312.	Outcomes of interest are not presented
Kilic N, Oktay H. Effects of rapid-slow maxillary expansion on the dentofacial structures. Aust Orthod J. 2010 Nov;26(2):178-83. PMID: 21175029.	Outcomes of interest are not presented
Ghoneima A, Abdel-Fattah E, Eraso F, Fardo D, Kula K, Hartsfield J. Skeletal and dental changes after rapid maxillary expansion: a computed tomography study. Aust Orthod J. 2010 Nov;26(2):141-8. PMID: 21175023.	Outcomes of interest are not presented
